# Biology of Disease Vectors, 2nd ed.

**DOI:** 10.3201/eid1108.050610

**Published:** 2005-08

**Authors:** Jerome Goddard

**Affiliations:** * Mississippi Department of Health, Jackson, Mississippi, USA;; † University of Mississippi Medical Center, Jackson, Mississippi, USA

**Keywords:** Vector biology, Insecticide Resistance, Entomology

This edition is a massive, 7-section, 57-chapter medical entomology reference text. The chapters are written by 72 experts from around the world and provide an understanding of disease vectors on a broad front, including biologic requirements of vectors, epidemiology, molecular biology, genetics, principles of control, and insecticide resistance. The text consistently emphasizes molecular biologic approaches to these topics.

This book begins by discussing the vectors themselves, with chapters on mites, ticks, true bugs, lice, fleas, mosquitoes, and various dipterans such as tsetse flies and sand flies. Line drawings and black-and-white pictures abound. The number of color photos is limited; those in the kissing bug/bed bug chapter and the flea chapter are especially beautiful. Subsequent sections delve into the physiologic and genetic basis of vector biology. The final 2 sections concern controlling insects and acarines and special (laboratory) methods associated with vectors. The last section, which deals with laboratory methods, is like a giant appendix in which updates are given for the care, maintenance, and experimental infection of various disease vectors, including notes on handling, housing, rearing facilities, containment, and safety issues.

One of the most helpful chapters for this reviewer was the one entitled, "Systematic Relationships among Disease Vectors," which defines molecular systematics terminology and explains how phylogenetic relationships among species are inferred from molecular data. I wish every traditional taxonomist and systematist would read this chapter.

This book contains a few misspellings (e.g., the chapter title in the Table of Contents, Chapter 46), but no major errors. Its only weakness seems to be one of disunity. The title doesn't match the book's content, and the text is so comprehensive that it seems unfocused. There are chapters on chemical and genetic control of vectors, cell culture, and even research safeguards for transgenic mosquitoes. How these fit under the title biology of vectors was difficult to discern. Perhaps in future editions, the chief editor could split the book into several separate volumes, each with a more appropriate title.

Nevertheless, this book is an indispensable reference and a wonderful treasure trove of information about medical entomology. Its only flaws are organizational, not factual. The chief editor, section editors, and authors are to be congratulated on this scholarly work.

